# Inflammatory Myofibroblastic Tumor of the Right Atrium

**DOI:** 10.1155/2010/695216

**Published:** 2010-09-19

**Authors:** Neerod K. Jha, Michel Trudel, Gregory P. Eising, Peter Lange, Awatif Al Sousi, Wael Al Mahmeed, Javed A. Khan, Moataz A. Saleh, Friederike Von Canal, Virendra K. Misra, Norbert Augustin

**Affiliations:** ^1^Division of Adult Cardiac Surgery, Sheikh Khalifa Medical City (Managed by Cleveland Clinic), P.O. Box 51900, Abu Dhabi, UAE; ^2^Division of Pathology, Sheikh Khalifa Medical City (Managed by Cleveland Clinic), P.O. Box 51900, Abu Dhabi, UAE; ^3^Division of Radiology, Sheikh Khalifa Medical City (Managed by Cleveland Clinic), P.O. Box 51900, Abu Dhabi, UAE; ^4^Division of Cardiology, Sheikh Khalifa Medical City (Managed by Cleveland Clinic), P.O. Box 51900, Abu Dhabi, UAE; ^5^Division of Anesthesiology, Sheikh Khalifa Medical City (Managed by Cleveland Clinic), P.O. Box 51900, Abu Dhabi, UAE

## Abstract

Cardiac inflammatory myofibroblastic tumor (IMT) is a rare entity and is associated with distinct clinical, pathological and molecular features. The clinical behavior, natural history, biological potential, management and prognosis of such tumors are unclear. We present herewith an adolescent girl who presented with similar entity involving the junction of the right atrium and the inferior vena cava (IVC) in association with thrombocytosis and IVC thrombosis leading to obstruction of blood flow. Diagnostic tools included imaging and immuno-histopathology studies. Surgical management included resection of the tumor and thrombo-embolectomy of the IVC under cardiopulmonary bypass. This case is unique due to association of complete obstruction of IVC caused by the strategic location of the tumor, thrombosis of vena cava and association of thrombocytosis. These features have not been reported yet in relation to the cardiac IMT. This report will help in better understanding and management of similar cases in terms of planning cannulation of femoral veins or application of total hypothermic circulatory arrest during cardiopulmonary bypass and prompt us to look for recurrence or metastasis during follow up using echocardiography and laboratory investigations. The possibility of IMT should be kept in the differential diagnosis of cardiac tumors especially in children and adolescents.

## 1. Introduction

Primary cardiac tumors are exceedingly rare in children and adolescents with a reported overall prevalence of 0.08% [1]. Majority of such tumors are benign which include rhabdomyoma, fibroma, myxoma or, teratoma. However, recently, Inflammatory Myofibroblastic Tumor (IMT) involving cardiac structures has emerged as a distinct entity with characteristic clinical, pathological, and molecular features such as a predilection for the visceral soft tissues with a tendency for local recurrence, fasciitis-like compact spindle cell and hypocellular fibrous pattern, and chromosomal translocation leading to activation of the ALK tyrosine kinase in almost 50% of the cases [1]. There are only few reports published describing IMT in patients between 4 months to 17 years of age with cardiac tissue involvement [2–7]. We are reporting herewith an adolescent girl who presented with an obstructive mass (IMT) within the right atrium-inferior venacava junction causing visceral congestion, thrombosis of the inferior venacava (IVC), and thrombocytosis, who underwent urgent surgical intervention. These clinical features are not previously reported for cardiac IMTs. Due to rarity, behavior, natural history, management, and prognosis of cardiac IMT are unclear. Therefore, this paper is not only an addition to the existing literature but also will help in better understanding and management of similar cases.

## 2. Case Presentation

A 14-year-old girl was referred to us for evaluation of a right atrial mass diagnosed incidentally in the referring hospital after an appendicectomy.

**Figure 1:**
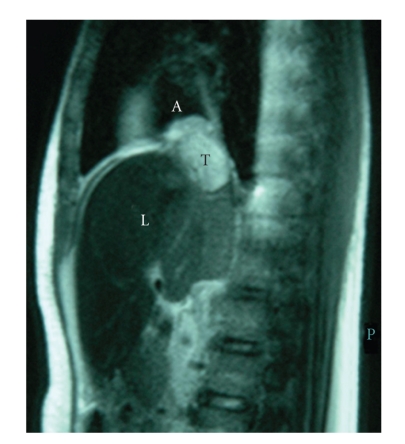
Magnetic resonance scan showing a large tumor mass (T) at the junction of the right atrium (A) and inferior vena cava in association with congested liver (L).

The patient had pedal oedema and hepatomegaly. The laboratory tests revealed leucocytosis, thrombocytosis, and elevated liver enzymes. Two-dimensional echocardiography, computer tomography including magnetic resonance imaging of the chest showed presence of right-sided contrast-enhancing mass in the right atrium near IVC junction with a size of 35 × 38 mm in cross-sectional dimensions (Figure 1). In addition, there was large thrombus in the IVC extending below the level of renal veins, causing liver congestion and ascitis. 

**Figure 2:**
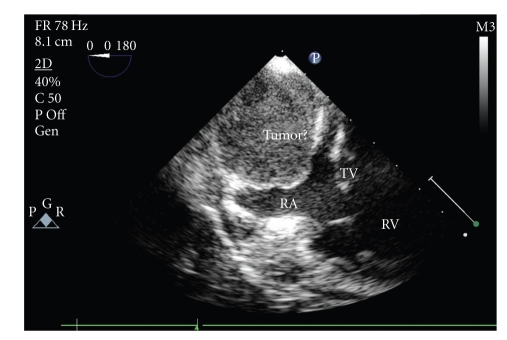
Transesophageal echocardiogram showing the homogenous echogenic mass (tumor) attached to the posterior wall of the right atrium (RA) above the tricuspid valve (TV), RV: right ventricle.

**Figure 3:**
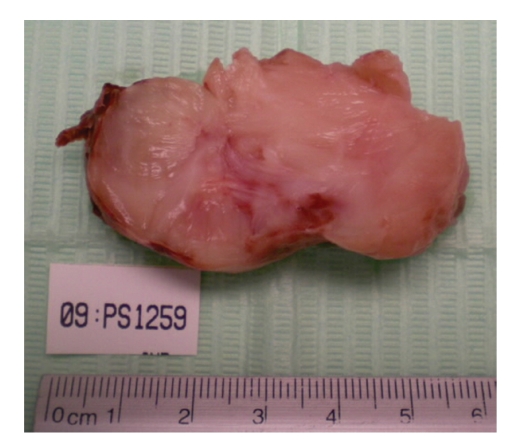
The cut section of excised tumor mass.

In view of the severe obstructive features, possibility of tumor embolization and unknown nature of the cardiac mass, an urgent surgical intervention was undertaken under standard cardiopulmonary bypass. Intraoperative transoesophageal echocardiography confirmed the location of tumor within the right atrium (Figure 2). The cannulation of ascending aorta, superior vena cava, and right common femoral vein was done. Under systemic hypothermia (25°C), aortic cross-clamping and cardioplegic arrest, the right atriotomy was performed. A large (5 × 5 cm) hard, grayish, nodular mass was found impacted at the IVC-right atrial junction circumferentially, and it was difficult to locate the exact point of attachment. The tumor was excised completely (Figure 3). A 4-minute of deep hypothermic circulatory arrest (20°C) was instituted to remove thrombus from the proximal IVC using Fogarty's catheter and saline flush. The postoperative course was uneventful. At 1-year follow-up the patient is asymptomatic and has no clinical or biochemical features of recurrence.

**Figure 4:**
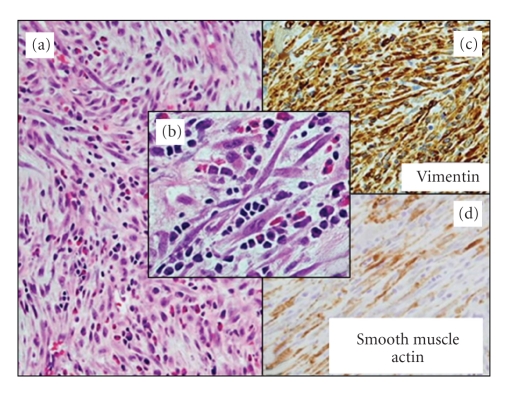
(a) and (b) Histopathology of tumor showing proliferation of myofibroblasts, inflammatory cells, spindle, and fusiform cells in a myxoid stroma (hematoxylin & eosin, x100 & x400 magnifications). (c) Immunophenotype—Vimentin expression in myofibroblastic population (immunoperoxidase, x200). (d) Immunophenotype—Smooth muscle actin coexpression (immunoperoxidase, x200)

Histopathology of the tumor revealed presence of myofibroblasts, fusiform and spindle cells, modest inflammatory infiltrates, and myxoid stroma in the background of small lymphocytes, plasma cells, and eosinophils (Figures 4(a) and 4(b)). The immunostain marker profile showed productivity for vimentin, smooth muscle actin isoforms, and MyoD, but negativity for desmin, ALK-1 protein, and S-100 protein (Figures 4(c) and 4(d)). The constellation of findings described above established a diagnosis of IMT.

## 3. Discussion

Usually, IMT involves lungs, liver, stomach, lymph nodes, retroperitoneal tissue, and spleen [1]. World Health Organization's classification has included IMT as tumor of intermediate biological potential due to tendency for local recurrence and small risk of metastasis [1]. Cardiac involvement is very rare [1–7]. In the available papers, involved cardiac structures were right atrium, right ventricle, tricuspid valve, and inter ventricular septum [2–7]. 

There are no specific signs or symptoms related to the cardiac IMT's, as these are related to the location within the heart. However, these tumors are thought to elaborate cytokines that may produce constitutional symptoms and signs such as fever, anorexia, anemia, hyper gamma-globulinemia, leukocytoclastic vasculitis, polyarthritis, and thrombocytosis [1, 3]. The pathogenesis of IMT is thought to be an exaggerated immunologic response by proliferated spindle cells and primary myofibroblasts to injury, inflammation, or infection [3]. In addition, the IMT is characterized by expression of vimentin, smooth muscle actin, and cytokeratins, corresponding to those of myofibroblasts along with other inflammatory markers [8]. In our patient the Interlukin-6 (IL-6) was also found to be elevated (17 ng/ml). Several authors have demonstrated production of IL-6 mRNA and protein by tumor cells, supporting a possible analogous mechanism in IMT. Interleukin production is thought to be an additional underlying cause of the systemic symptoms and complications which may return to normal postoperatively [1]. Similarly, IL-6 and cytokines may be inflammatory markers of tumor recurrence during postoperative follow-up [1].

Surgical excision of the tumor has been the mainstay of the management, though radiotherapy, immunosuppressant, and chemotherapy have been tried as an adjunct to surgery without additional benefit [8]. In our case, due to obstruction of IVC caused by tumor and associated thrombosis, an urgent surgical intervention was warranted. Moreover, complete removal of thrombus from the suprahepatic IVC required cannulation of right common femoral vein and a brief period of circulatory arrest. Therefore, a careful observation, planning, and timely surgical management is the key to the successful outcome in similar cases.

## 4. Conclusion

Cardiac IMT's are potentially benign lesions with favorable prognosis and should be considered in the diagnosis of cardiac tumors in children and adolescents. Surgical management is currently the preferred treatment approach. Although, recurrence or metastasis of cardiac IMT has not been reported yet, the followup of such patients should be aimed to monitor tumor recurrence which may be heralded by a return of clinical or laboratory abnormalities. Therefore, a physical examination, echocardiography, and blood tests for inflammatory markers, platelets count, and IL-6 levels are mandatory at regular intervals during long-term follow-up. In addition, patients should be informed about variable biological behavior and nature of this entity.
